# Parents and prevention research - is it possible? A systematic review of the literature

**DOI:** 10.1186/2050-2974-1-S1-O39

**Published:** 2013-11-14

**Authors:** Susan Paxton, Chelsea Cornell

**Affiliations:** 1 La Trobe University, Australia

## 

Parents have been identified as playing an important role in promoting either risk or protective factors for the development of body image or eating problems in their children. The purpose of this study was to systematically review the literature on eating disorder prevention programs that include a component for parents. A range of academic databases were searched for English language publications between January 1992 and April 2012. 443 novel records were screened for eligibility. Studies needed to meet three inclusion criteria: 1) Delivery of a prevention program designed to reduce eating disorder or body image dissatisfaction in children (2) Some program component specifically targeted to parents (3) Implementation and reporting of an outcome measure to evaluate the prevention program. 18 papers were included in the review. Although three studies attempted to compare parental involvement with student-only interventions, very small sample sizes and a lack of parental engagement hampered statistical analyses. The majority of studies incorporated a minimal parental component in school-based interventions for pre- or young adolescents, without thorough evaluation of impact or effects. While research involving parents is well developed in prevention of obesity and externalising disorders, there are significant gaps in the eating disorders field. Researchers need to focus on effective parental engagement and rigorous evaluation design before parents can be optimally involved in prevention research.

This abstract was presented in the **Prevention** stream of the 2013 ANZAED Conference.

